# *Caenorhabditis elegans* AWC neuron-mediated chemosensation negatively modulates dormancy during *Salmonella fepB* mutant infection

**DOI:** 10.1128/spectrum.00420-25

**Published:** 2025-10-08

**Authors:** Swarupa Mallick, Jasmin Pradhan, Chamjailiu Daimai, Vidya Devi Negi

**Affiliations:** 1 Infection Immunology Laboratory (2i-Lab), Department of Biological Sciences, Indian Institute of Science Education and Research (IISER) Mohali, Knowledge City, Punjab, India; 2 Laboratory of Infection Immunology, Department of Life Science, National Institute of Technology 29737, Rourkela, Odisha, India; National University of Singapore, Singapore, Singapore

**Keywords:** *Salmonella *Typhimurium, *Caenorhabditis elegans*, dauer, AWC, *fepB*

## Abstract

In our earlier work, we demonstrated that continuous 8-day exposure of *Caenorhabditis elegans* to a *Salmonella enterica* serovar Typhimurium *fepB* mutant strain negatively regulates dauer larva development in the second generation of the population. Our current study aims to understand how specific chemosensory neurons in *C. elegans* recognize the *∆fepB Salmonella* Typhimurium strain and undergo plasticity in response to infection. We observed the olfactory preference of *C. elegans* toward the pathogenic wild type *Salmonella* (WT-STM). However, prolonged exposure showed enhanced lawn occupancy of nematodes in the ∆*fepB* strain with better associative learning response than the WT-STM counterpart. We also observed upregulation of chemosensory genes *odr-7*, *ceh-36*, *daf-11*, *tax-2*, and *tax-4* at 24 hours post ∆*fepB* infection. However, continuous exposure to defective olfactory neuron mutants of the *C. elegans* emphasizes AWC neurons’ participation in sensing the Δ*fepB* strain, ultimately mediating plasticity in *C. elegans*’ second generation. Our research shows how the olfactory neurons of *C. elegans* detect *Salmonella* Typhimurium upon encounter and adjust their behavior accordingly. Furthermore, it highlights the strong connection between the chemosensory neurons of nematodes and the bacterial signals that regulate host physiology for survival.

**IMPORTANCE:**

Bacteria act as food signals for the *Caenorhabditis elegans*. Our work gives insight into how worms’ olfactory neurons recognize pathogen *Salmonella* Typhimurium exposure and modulate behavioral plasticity, giving a better survival strategy against the pathogens. How specific chemosensory neurons in worms recognize the ∆*fepB* strain and undergo behavioral plasticity in response to infection. Furthermore, it highlights the strong connection between the chemosensory neurons of worms and the bacterial signals that regulate host physiology for survival when exposed to mutant strain infection, which might be under check in wild-type bacteria for their own benefit in an evolutionary adaptation. This mechanism might help the worm to select the pathogenic or non-pathogenic microbes as food and avoid infection-mediated lethality.

## INTRODUCTION

Food is necessary for *Caenorhabditis elegans* and any organism’s optimum fitness and survival (1). However, predation or pathogen attack is common for them while searching for food in their niches. *C. elegans* is a free-living soil nematode commonly found in complex environments (decaying vegetation, rotten fruits, and compost) in close association with pathogenic and non-pathogenic microorganisms, building a hostile situation for optimum survival. However, *C. elegans* has a well-developed chemosensory system with 302 neurons that enable it to locate food and sense danger or other animals in their natural habitat (2, 3). Such chemosensory neurons will translate the chemical cues perceived from their environment and initiate neuroendocrine signaling, allowing nematodes to discriminate between edible and pathogen-contaminated food, drive physical avoidance behaviors, and influence their developmental choices (4, 5).

*Salmonella* Typhimurium 14028s strain (WT-STM), a Gram-negative, facultative anaerobic bacterium, is known to cause persistent infection in *C. elegans* and lead to their death (6, 7). Our earlier study found that infecting *C. elegans* with the ∆*fepB S*. Typhimurium strain resulted in a significant increase in dauer larvae development, an alternative third larval stage, in their second generation compared to WT-STM infection. This phenotype persisted until the fourth generation, under continuous infection with the pathogen. In *Salmonella*, the *fepB* gene is part of an ABC transporter (FepBDGC operon) involved in iron uptake, particularly ferric iron. It participates in transporting ferric enterobactin, a siderophore that helps the bacteria obtain iron from their environment with limited iron availability, like host cells. Deletion of this gene disrupts the iron acquisition and leads to reduced persistence, enhanced host immune response, and better survival ability of *C. elegans*, which was observed in our study. We also infected the *C. elegans* with ∆*ftnB* and ∆*bfr Salmonella* strains (ferritin family genes essential for iron storage, intracellular survival, and virulence of *S*. Typhimurium) to detect the direct or indirect involvement of iron in nematodes’ dauer larvae development but did not observe an elevated level of dauer larvae as compared to ∆*fepB*. We finally found out that the *fepB* gene in WT-STM negatively regulates dauer larvae development upon continuous infection through the TGF-β signaling pathway, emphasizing that food provides signals that modulate the normal physiology of nematodes during infection (8–10). However, the question remains: how does *C. elegans* recognize the ∆*fepB* strain that affects its normal development? Our current study explores the mechanisms behind the nematodes’ chemosensory response to the Δ*fepB* strain and how this chemosensation influences their developmental decisions during infection. Our study on bacteria-emitted signals and organism physiology helps understand complex host-pathogen interactions in *Salmonella* pathogenesis, which can later be explored in higher organisms.

## RESULTS

### *Salmonella fepB* mutant infection altered *C. elegans* behavior

**Fig 1:**
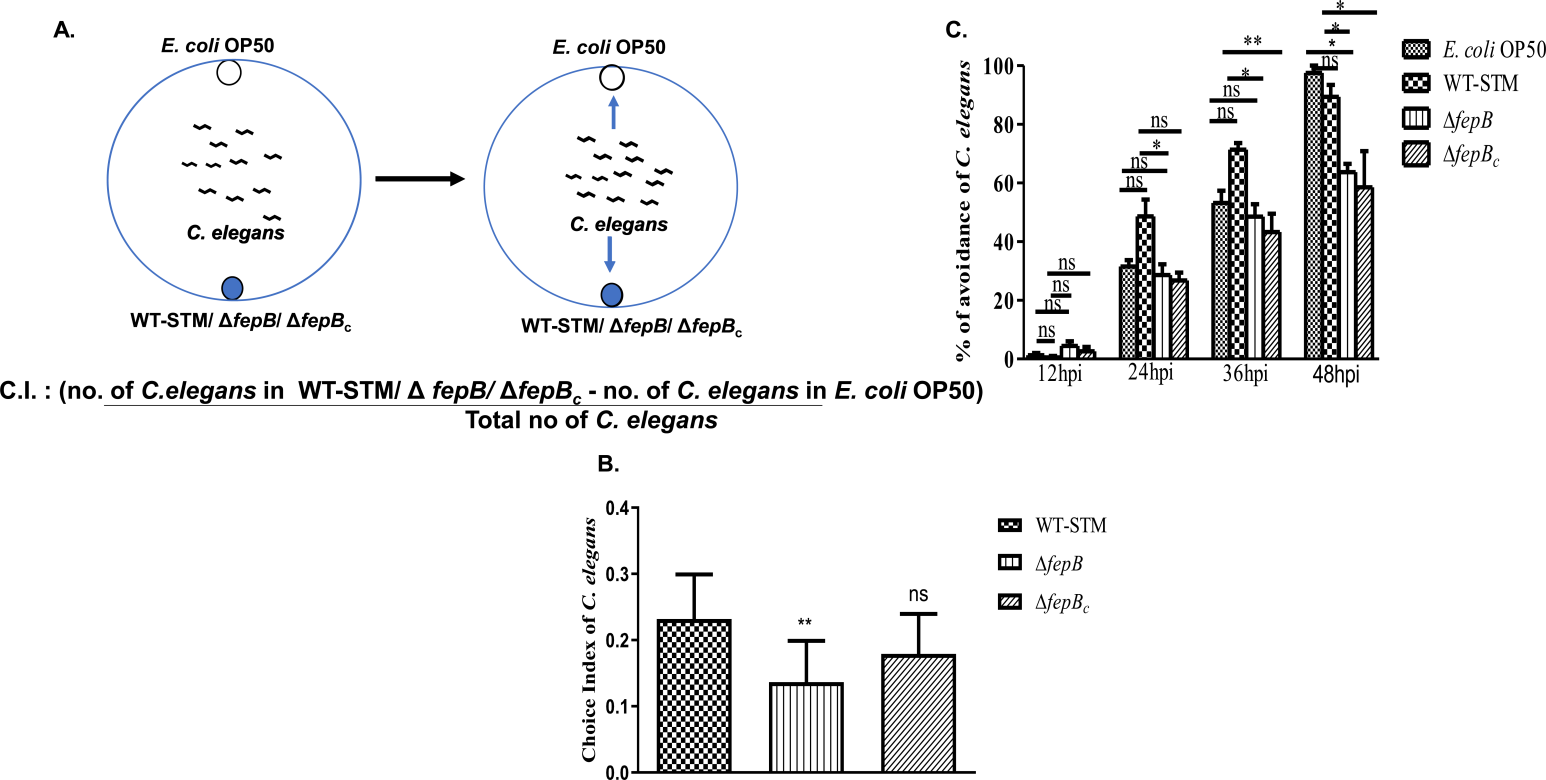
*C. elegans* exhibited altered chemosensation against the *Salmonella fepB* mutant strain. (**A**) Schematic presentation of binary food choice assay where age-synchronized one-day adult *C. elegans* were placed in the middle of NGM plates seeded with *E. coli* OP50 at one side of the plate and *S*. Typhimurium (WT-STM)/Δ*fepB/*Δ*fepB_c_* on another side of the plate. *C. elegans* were kept under these conditions for 1–2 hours and observed their food preference. (**B**) Quantitative analysis of *C. elegans* food preference toward WT-STM/Δ*fepB/*Δ*fepB_c_* was plotted and shown in the bar. (**C**) Quantitative analysis of *C. elegans* avoidance response was plotted and shown in the bar. The experiments represented three biological replicates with three technical replicates, and the result significance was quantified as *P*-value < 0.05 ∗, *P*-value < 0.005 ∗∗, and *P*-value < 0.0001 ∗∗∗, with ns-non-significant values expressed as mean ± SEM.

*C. elegans* can recognize their pathogens and mount specific protective responses like avoidance, resistance, or secretion of antimicrobial peptides (AMPs), reactive oxygen species (ROS), and inducible nitric oxide synthase (iNOS) for optimum survival under hostile conditions (5, 11). Our previous study demonstrated that ∆*fepB* infection caused behavioral plasticity in the second generation, while giving better viability under infection conditions than WT-STM (10). This led us to investigate the *C. elegans* behavior in the first generation of the total population during infection. To address this query, we observed the nematode’s behavior, i.e., *C. elegans* food preferences over 1–2 hours time intervals under infection conditions, aiming to understand their olfactory preference. We discovered that nematodes showed a strong choice for WT-STM but did not prefer the ∆*fepB* strain (Fig. 1A and B). Next, we wanted to explore *C. elegans* behavior under prolonged infection conditions. We found that continuous exposure altered the behavioral responses in *C. elegans*, with increased Δ*fepB* strain lawn occupancy compared to the WT-STM lawn (Fig. 1C). Overall, the behavioral study implied that WT-STM lures *C. elegans*, but continuous exposure exhibited an increased lawn aversion from the WT-STM lawn to the Δ*fepB* lawn.

### *C. elegans* showed enhanced olfactory function toward the Δ*fepB Salmonella* strain

**Fig 2:**
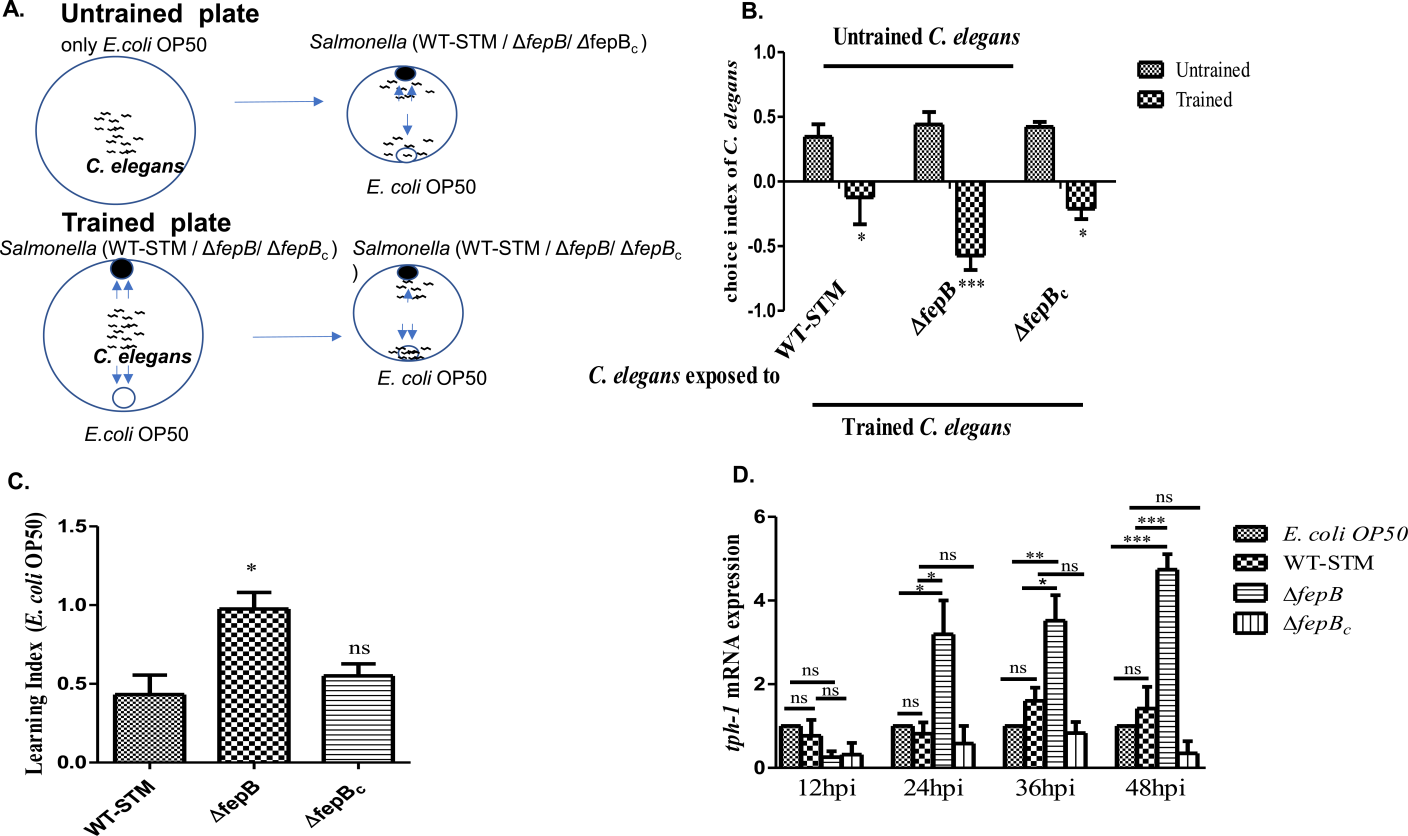
Enhance olfactory function against the Δ*fepB Salmonella* strain in *C. elegans*. (**A**) Schematic representation of *C. elegans* olfactory aversive learning behavior, where age-synchronized L4 *C. elegans* larvae were kept in an *E. coli* OP50 plate (naïve plate/untrained plate) and in NGM plates seeded with *E. coli* OP50 at one side and WT-STM/ Δ*fepB/* Δ*fepB_c_* on another side of the plate (test plate/trained plate). *C. elegans* were kept under these conditions for 48 hours. Then, *C. elegans* from both untrained and trained plates were taken, washed 3–4 times, and placed on an assay plate containing *E. coli* OP50 on one side and *S*. Typhimurium (WT-STM)/Δ*fepB/*Δ*fepB_c_* on the other side of the plate. After 1–2 hours, the *C. elegans* aversive learning behavior was observed. (**B**) Quantitative analysis of the choice index of the *C. elegans* under-trained and untrained conditions was plotted and shown in the bar. Here, +1 represents *C. elegans* preference toward WT-STM and mutant strains, −1 represents preference toward *E. coli* OP50, and 0 represents the equal preference of *C. elegans* toward *E. coli* OP50, WT-STM, and its mutants. (**C**) Quantitative analysis of the learning behavior of *C. elegans* was plotted and shown in the bar. Age-synchronized L4 worms were kept in *E. coli* OP50, WT-STM/Δ*fepB/*Δ*fepB_c_* plates for 12, 24, 36, and 48 hours. *C. elegans* were taken for RNA isolation. (**D**) Quantitative real-time PCR (qRT-PCR) was performed for *tph-1* genes expression, using *ama-1* as an endogenous control, and shown as relative fold changes calculated by using the comparative 2^ΔΔCT^ method. Data represent three biological replicates and three technical replicates, result significance quantified as *P*-value < 0.05 ∗, *P*-value < 0.005 ∗∗, and *P*-value < 0.0 0 01 ∗∗∗, with ns-non-significant. Values expressed as mean ± SEM.

Many reports have revealed that *C. elegans* can recognize and remember their pathogens for survival and fitness under that condition (12–17). Pathogenic bacteria, e.g., *Salmonella enterica*, *Serratia marcescens*, *Pseudomonas aeruginosa*, and *Bacillus thuringiensis*, induce nematodes to develop aversive learning behavior through the altered feeding preference toward non-pathogenic *Escherichia coli* OP50 (11, 12). To test the ability of *C. elegans*’ to recognize pathogens, we exposed nematodes in two different conditions: (i) a naïve condition, in which *C. elegans* were placed in an *E. coli* OP50-seeded nematode growth media (NGM) plate, and (ii) a trained condition, in which *C. elegans* were on an NGM plate containing *E. coli* OP50 seeded at one side and WT-STM/Δ*fepB*/Δ*fepB_c_* on the other side of the plate and incubated for 48 hours to acclimatize to the odor of these pathogens (Fig. 2A). Here, we observed that *C. elegans* exposed to the Δ*fepB* strain for 48 hours exhibited significantly stronger learning behavior than the WT-STM (Fig. 2B and C). Various reports have revealed that, in *C. elegans*, *tph-1* (tryptophan hydroxylase, a rate-limiting enzyme involved in serotonin biosynthesis) plays a vital role in various neural functions, and its expression is highly regulated by environmental conditions, i.e., starvation, food quality, and temperature. Experience-dependent changes in *tph-1* expression are found under pathogen exposure (18–20). In our study, we also observed that *C. elegans* infected by the Δ*fepB* strain exhibits an upregulated *tph-1* expression in a time-dependent manner compared to WT-STM (Fig. 2D), which implied enhanced olfaction and better learning behavior of *C. elegans* against the mutant *Salmonella* strain.

### AWC-mediated chemotaxis in *C. elegans* against the Δ*fepB* strain

**Fig 3:**
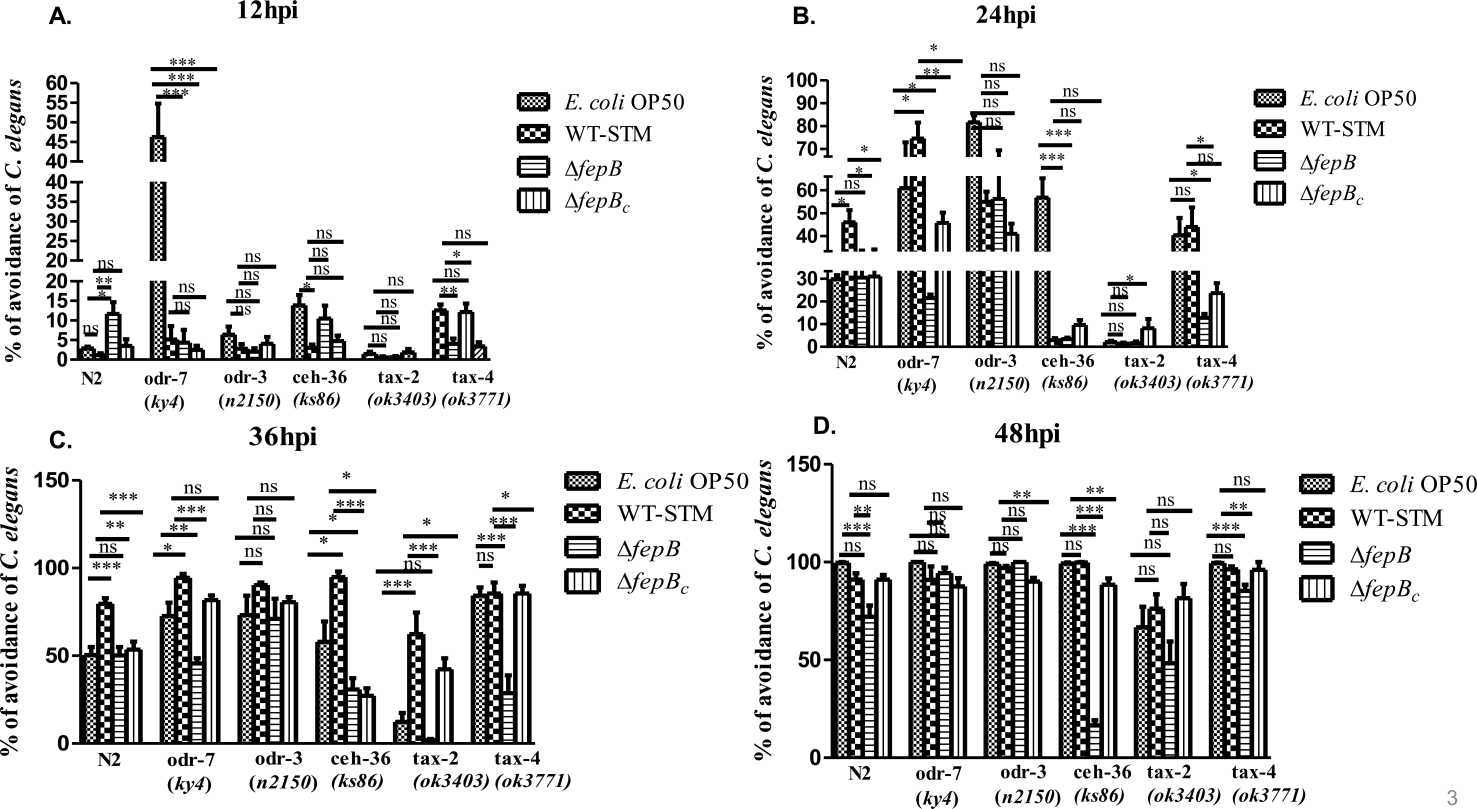
*C. elegans ceh-36* has a role in sensing the *fepB* mutant *Salmonella* strain. Age-synchronized one-day adult *C. elegans* (N2, *odr-7*, *odr-3*, *ceh-36*, *tax-2*, and *tax-4*) were exposed to *E. coli* OP50, WT-STM, Δ*fepB*, and Δ*fepB_c_,* and avoidance behavior in *C. elegans* in a time-dependent manner was observed. (**A−D**) Quantitative analysis of *C. elegans* avoidance response was plotted and shown in the bar, and the results were compared to those of N2 worms. Data represented three biological replicates and three technical replicates; result significance was quantified as *P*-value < 0.05 ∗, *P*-value < 0.005 ∗∗, and *P*-value < 0.0001 ∗∗∗, ns: non-significant. Values expressed as mean ± SEM.

**Fig 4:**
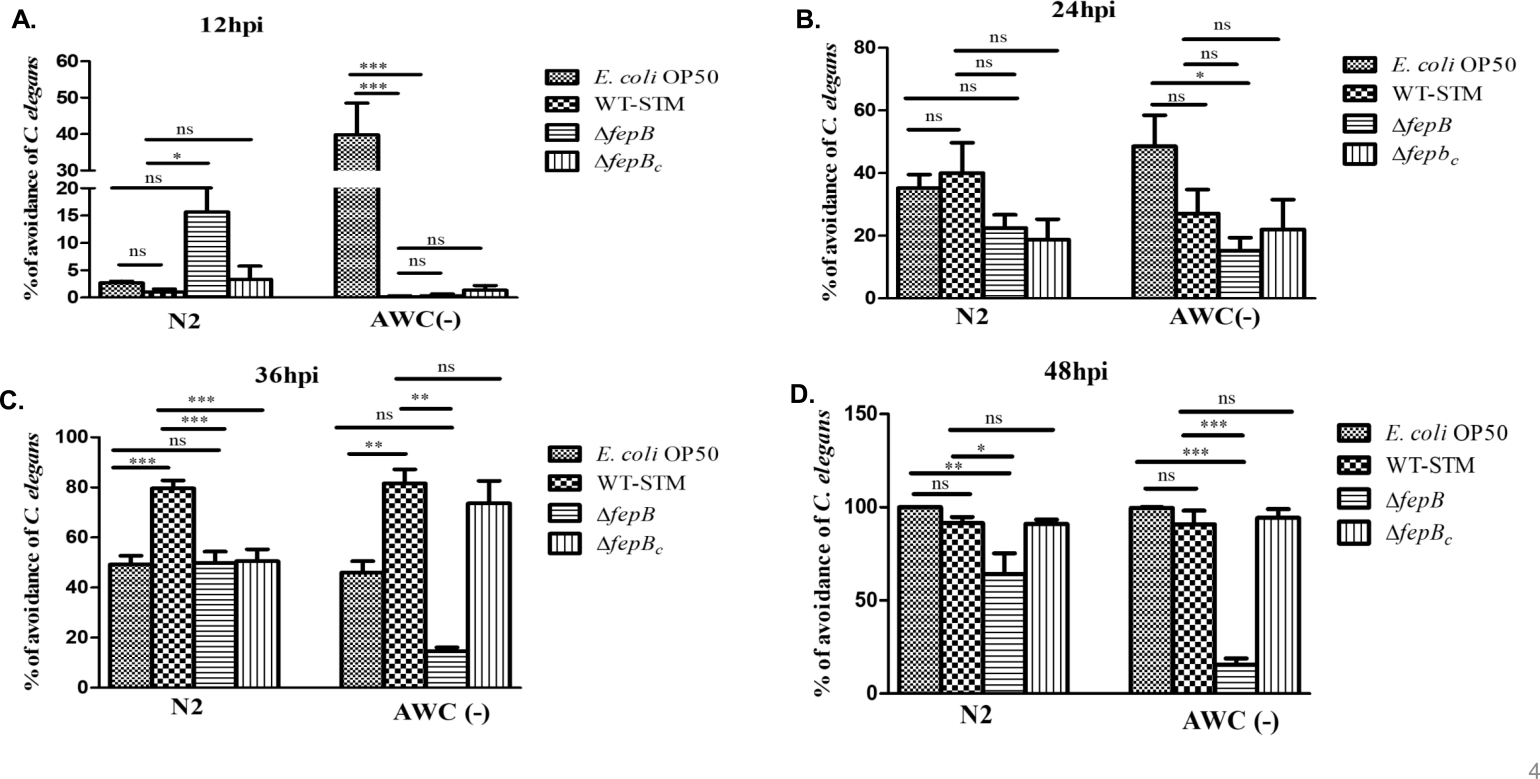
*C. elegans* AWC neurons participate in *fepB* mutant *Salmonella* chemosensation. Age-synchronized one-day adult *C. elegans* (N2, AWC [−]) were exposed to *E. coli* OP50, WT-STM, Δ*fepB*, and Δ*fepB_c_* and observed avoidance behavior in *C. elegans* in a time-dependent manner. (**A−D**) Quantitative analysis of *C. elegans* avoidance response was plotted and shown in the bar; the results were compared to those of N2 worms. Data represent three biological replicates and three technical replicates; result significance was quantified as *P*-value < 0.05 ∗, *P*-value < 0.005 ∗∗, and *P*-value < 0.0001 ∗∗∗, with ns-non-significant. Values expressed as mean ± SEM.

*C. elegans* has shown a robust olfactory preference for WT-STM over 1–2 hours, indicating chemosensory neurons’ involvement in pathogen recognition. Three olfactory sensory neurons, amphid wing A, B, and C (AWA, AWB, and AWC) play a significant role in *C. elegans* olfaction (4, 5). To confirm the significant participation of any of these specific neurons in sensing bacterial strain, we first checked the mRNA expression of specific genes playing an important role in olfactory neuron-mediated olfaction in a time-dependent manner. For example, *odr-10*, which encodes a G-protein-coupled receptor expressed exclusively in AWA neuron, and ODR-10 is localized at the tip of the AWA ciliary ending required for AWA-mediated chemosensation, particularly for sensing diacetyl, as this ODR-10 is the receptor for diacetyl, and *odr-10* mutant *C. elegans* display AWA-defective response toward diacetyl (4, 21, 22). *odr-3* encodes the G protein α subunit and acts downstream of many olfactory and nociceptor receptors. A mutation in the *odr-3* gene exhibited defective responses mediated by AWA, AWB, AWC, and amphid single cilium H (ASH) neurons in *C. elegans* (21, 23–25). CEH-36 is a transcription factor playing an essential role in the terminal differentiation of AWC neurons, and mutation to this gene leads to defective responses to AWC-sensed odorant (26, 27). Besides, we also checked mRNA expression of the *daf-11* gene required for the AWB and AWC neuron-mediated olfactory behavior; it was reported that AWC neurons utilized a major signal transduction pathway through the DAF-11 guanylyl cyclase pathway, and mutation in the *daf-11* gene in *C. elegans* exhibited defective responses toward AWC-sensed odorant (21, 28, 29). However, most chemosensory neurons use cyclic nucleotide-gated ion channels or transient receptor potential (TRP) channels for sensory transduction. Cyclic nucleotide-gated channel encoded by *tax-2* and *tax-4* genes and *osm-9* and *ocr-2* encoding TRP channel for chemosensory signal transduction (24) (Table 2). Here, we observed a differential gene expression pattern, i.e., upregulation of *odr-10*, *ceh-36*, *daf-11*, and *tax-2/tax-4* genes at 24 hours post-infection against the Δ*fepB* strain, stating a strong involvement of chemosensory neurons for sensing the *Salmonella* strain (Fig. S1 and S2). Next, when we performed a lawn aversion response with nematodes having defects in olfactory neurons, i.e., AWA, AWB, and AWC, we found that with the increase in time, *ceh-36* mutant of *C. elegans* remained in the Δ*fepB* strain lawn as compared to the WT-STM lawn (Fig. 3A through D), indicating *ceh-36* (AWC neuron-specific gene) is involved in sensing the mutant *Salmonella* strain. To further confirm the significant involvement of AWC neurons in recognizing the *fepB* mutant strain, we exposed AWC (−) nematodes to infection conditions and noticed that at 36 and 48 hours post-infection, the AWC (−) strain was unable to show lawn aversion behavior against the *fepB* mutant strain (Fig. 4A through D). Overall, our data implied the role of AWC neurons in *C. elegans* chemosensation toward *Salmonella*, particularly recognizing the *fepB* mutant *Salmonella* strain under continuous infection conditions.

### AWC neuron involved in developmental plasticity of *C. elegans* against the Δ*fepB* strain

**Fig 5:**
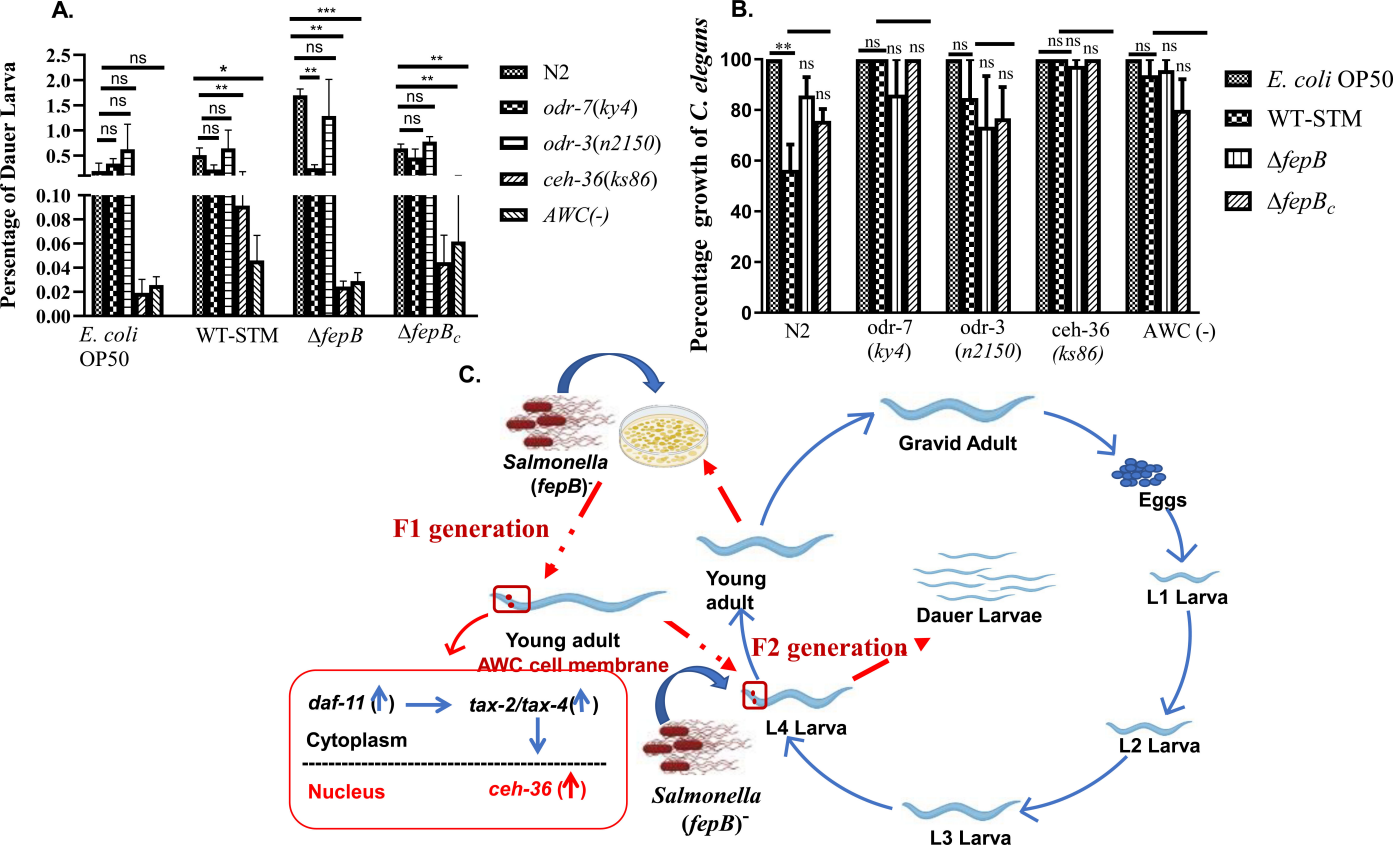
Δ*fepB* strain modulated dauer larva development in *C. elegans* through AWC-mediated chemosensation. Age-synchronized 10 L4 larvae (N2, *odr-7*, *odr-3*, *ceh-36*, and AWC [−]) were placed on the NGM plates seeded with non-pathogenic *E. coli* OP50, pathogenic WT-STM, Δ*fepB,* and Δ*fepB_c_* and kept for 8 days at 22°C. On day 8, dauer larvae were isolated using 1% SDS. (**A**) Quantitative analysis of dauer larvae development under control (*E. coli* OP50) and with infection (WT-STM and its mutant strains) conditions was plotted and shown in the bar. Dauer larvae formed under different mutant strains were compared to N2 worms. (**B**) Quantitative analysis of *C. elegans* strains (N2, *odr-7*, *odr-3*, *ceh-36*, and AWC [−]) growth on day 8. (**C**) Schematic representation of AWC neuron involvement for recognizing *Salmonella* strains in the F1 generation of *the C. elegans* population and modulating dauer larvae development under continuous infection conditions in their F2 generation. Data represented three biological replicates and three technical replicates; result significance was quantified as *P*-value < 0.05 ∗, *P*-value < 0.005 ∗∗, and *P*-value < 0.0001 ∗∗∗, ns-non-significant. Values expressed as mean ± SEM.

Exposure to stressful environmental stimuli, such as high temperatures, pheromones, food scarcity, and overcrowding, triggers the formation of an alternative third larval stage called the dauer, which enables nematodes to survive harsh conditions. Pathogens also serve as stressors and can influence nematode developmental plasticity (2, 10, 30–32). The molecular mechanism behind the dauer phenomenon is well understood, but the role of chemosensory neurons in dauer larvae under *Salmonella* infection requires further study. From the *C. elegans* behavior study described before, it was clear that AWC neurons played a significant role in sensing the Δ*fepB* strain. Thereafter, we wanted to understand the involvement of this neuron in modulating *C. elegans*’ plasticity. We examined dauer larvae development under control and infection conditions to answer this query using the olfactory defective mutant and N2 nematodes. Interestingly, we observed reduced dauer larvae development by *ceh-36* and AWC (−) *C. elegans* in the second generation of their population. However, the total population was not altered under the *fepB* mutant *Salmonella* strain infection condition (Fig. 5A and B). Overall, our study elucidates the involvement of chemosensory neurons, particularly AWC, in recognizing the *Salmonella fepB* mutant strain during the initial time of exposure. This recognition likely occurred via activation of the *daf-11* and *tax-2/tax-4* signaling pathway. Upon prolonged exposure, these neurons appear to contribute to behavioral plasticity. However, the specific mechanism by which AWC neurons detect the *fepB* mutant, whether through a secreted bacterial effector molecule or the FepB protein itself, remains to be clarified in future studies.

Here, Fig. 5C is a schematic representation indicating the significant participation of the *C. elegans* AWC neuron in sensing the *fepB* mutant *Salmonella* strain in the first generation and altered behavioral responses leading to the induction of dormancy in the *C. elegans* second generation against the Δ*fepB Salmonella* strain.

## DISCUSSION

*C. elegans* live in a complex environment that enables them to evolve with specific mechanisms to recognize and mount molecular and behavioral defense responses for optimum survival (12). Feeding different microorganisms, either pathogenic or non-pathogenic, has a different effect on the *C. elegans* life cycle. The type of food source can alter the nematode’s normal behavior and developmental process (30).

Reports show that Gram-negative bacteria, *P. aeruginosa*, infect and kill *C. elegans* either through slow killing by colonization and proliferation in the intestine or by secreting toxic metabolites, leading to the fast killing of the *C. elegans* (33). *S. marcescens*, another Gram-negative opportunistic human pathogen, was reported to cause a distended intestine in *C. elegans* and lead to death after 6 days of exposure (34, 35). An extensive study was conducted with *C. elegans* to understand different aspects of interaction with *S*. Typhimurium, another potent Gram-negative human pathogen known to cause gastroenteritis in humans (36). *S*. Typhimurium is a well-known pathogen that causes persistent infection in the *C. elegans* intestine, leading to nematode death (7, 36). Infecting *C. elegans* with *S*. Typhimurium strains showed persistence in the intestines, affecting their lifespan and ultimately leading to death (36, 37). Such bacteria also form static aggregates mediated by biofilm formation that can aid their prolonged survival *in vivo* by suppressing the host immune response (38). A mutant *Salmonella* strain that is less virulent in the mammalian system also showed attenuated virulence in *C. elegans* (7). Infection of *C. elegans* with *S*. Typhimurium revealed a germline developmental defect (39). Our earlier study demonstrated that deleting the *S*. Typhimurium *fepB* gene altered *C. elegans* physiology and induced dauer larvae development in their second generation under continuous infection for 8 days at 22°C. Besides, we observed better survival ability in nematodes infected by the Δ*fepB* strain, significant bacterial clearance from the *C. elegans*’ intestine, and even enhanced early innate immune response compared to the WT-STM counterpart, indicating less virulence property of the Δ*fepB* strain (10). The *fepB* gene plays a vital role in *Salmonella* pathogenesis. Studies by Palacios et al. and Zhang et al. have reported the importance of the *fepB* gene in pathogens’ virulence. In *Klebsiella pneumoniae*, deletion of the *fepB* gene showed more attenuated virulence properties than wild-type bacteria. In *E. coli*, the *fepB* mutation affecting the ferric enterobactin import system and feeding such bacteria to *C. elegans* caused oxidative stress that impairs mitochondrial function and delays nematode development (40, 41).

*C. elegans* develops a unique sensorimotor system to navigate its habitat and respond to various environmental cues, including odorants, salt, temperature, pheromones, gases, mechanical stimuli, etc. (11, 19, 42). To withstand pathogens’ attack, nematodes often employ a flight response, showing avoidance or fight response by secreting antimicrobial peptides, i.e., AMPs, iNOS, or ROS, for better survival. Frequently, these avoidance or aversive learning behaviors are one of the energy-sparing survival strategies for the *C. elegans* against pathogens and decrease the chance of infection (43, 44). Certain groups like Zang et al., Kaletsley et al., Sengupta et al., and Im Choi et al., reported that *C. elegans* in their environment showed avoidance against the odor secreted from pathogens and moving toward familiar non-pathogenic bacterial odorant; particular bacteria elicit specific changes in olfactory preferences in nematodes, leading to the associative learning behavior by simply avoiding the same pathogenic exposure for the second time. Also, small ribonucleic acid (sRNA) from pathogenic bacteria, such as *P. aeruginosa*, *P. vranovensis*, etc., can modulate avoidance behavior in *C. elegans* and pass the information to their progeny for better survival (12, 16, 17, 45). From our initial study, we were curious to understand how this Δ*fepB* strain affects nematodes’ physiology in the first generation, along with their chemosensation toward the Δ*fepB* strain and developmental plasticity in their second-generation upon continuous infection. Using a binary food choice assay, we first examined the *C. elegans* behavior toward the bacterial pathogen. The volatile odor released by bacteria predominantly determines the *C. elegans’s* initial approach (5). We observed altered behavior of *C. elegans* where they significantly moved away from the Δ*fepB* strain compared to the WT-STM counterpart, showing an initial strong olfactory preference toward WT-STM (Fig. 1A and B). However, such olfactory preference against *fepB Salmonella* mutant strains was changed in *C. elegans* under prolonged exposure, which could modulate their behavioral responses (Fig. 1C). Since such study was deciphering nematodes’ strong preference for the WT-STM, we were interested in understanding *C. elegans*’ ability to remember and recognize this pathogen. Studies have revealed the nematodes’ olfactory aversive learning behavior, where the nematodes can avoid a pathogen while exposed to the same for the second time (12–17). In our study, after giving nematodes 48 hours of training with non-pathogenic *E. coli* OP50, pathogenic WT-STM, and its mutant strains Δ*fepB*, the nematodes showed an increased associative learning behavior against the Δ*fepB* strain than the WT-STM counterpart, and the complement strain for *fepB* displayed restoration of phenotypes (Fig. 2A through C). Besides, an upregulation of the *tph-1* gene (required to synthesize serotonin in the *C. elegans* ADF neuron that provides olfactory aversive learning behavior in *C. elegans* [12, 13, 19]) was observed in a time-dependent manner against the Δ*fepB* strain than wild-type *Salmonella* (Fig. 2D). The behavior study data implied nematodes’ strong ability to judge food quality, leave the pathogenic bacterial lawn, and develop associative learning behavior by taking the non-pathogenic diet. This finding led us to explore the involvement of *C. elegans* chemosensory neurons, particularly olfactory neurons, in recognizing these bacterial strains and showing the observed phenotype.

*C. elegans* possesses well-developed chemosensory neurons to sense pathogen-secreted odors that are attractive or repulsive. Three olfactory chemosensory neurons in the nematodes’ head region participate in sensing volatile odors in their vicinity. Neurons that mainly detect water-soluble cues (gustatory neurons) show distinct morphology from those sensing volatile (olfactory neurons) cues from the environment (24, 46). Gustatory neurons, i.e., amphid single cilium E, G, H, I, J, K (ASE, ASG, ASH, ASI, ASJ, ASK) and amphid dual ciliated ending F and L (ADF, L) have a simple single or double ciliary ending in their amphid opening with direct contact to the environment. Three pairs of amphid sensory neurons, i.e., AWA, AWB, and AWC, can detect their environment’s volatile chemical cues. These neurons have elaborated ciliated sensory endings in a sheath cell near the amphid opening with a large surface area to detect optimum volatile odorant. AWA and AWC allow for the detection of attracted odorants, whereas AWB senses repellent (5, 35, 47). We initially checked the mRNA expression of specific chemosensory genes (*odr-10*, *odr-3*, *ceh-36*, and *daf-11*) known to play an essential role in chemosensory neurons AWA, AWB, and AWC mediated olfaction, along with sensory transduction genes (*tax-2*, *tax-4*, *osm-9*, and *ocr-2*; Table 2) (21–23, 25, 29) in a time-dependent manner. We observed the differential gene expression in the concerning nematode at various post-infection time points following exposure to the *fepB* mutant bacterial strain (Fig. S1 and S2). These expression changes varied over time, suggesting the involvement of the targeted neuron in the host response.

To further validate the strong involvement of the chemosensory neurons in recognizing the *fepB* mutant *Salmonella* strain and modulating nematodes’ behavior toward the mutant strain, we used *C. elegans* mutants (*odr-7*, *odr-3*, *che-36*, and *tax/2/4*) with defects in their olfactory neurons. We found that in a time-dependent manner, *ceh-36* mutant nematodes failed to respond to the *fepB* mutant *Salmonella* strain even at 48 hours of continuous infection, which emphasized that AWC neurons might participate in sensing the *Salmonella* mutant strain, as the Ceh-36 transcription factor aids terminal differentiation of AWC neurons and leads to AWC-mediated olfaction in *C. elegans* (Fig. 3A through D). To confirm the strong involvement of AWC neurons, we exposed AWC-ablated *C. elegans* to similar infection conditions with all bacterial strains. We checked an aversive response against the Δ*fepB* strain. Here, mutant *C. elegans* could not show the lawn avoidance behavior against the said pathogen at a later time of infection, supporting our observation that the AWC olfactory neuron plays an important role in sensing the Δ*fepB* strain (Fig. 4A through D).

With the changing environmental conditions, *C. elegans* must also alter its development and behavioral programs. The expression and flexible modulation of specific chemosensory genes are vital for shaping the particular behavior of *C. elegans*. However, modulation of these highly dynamic chemosensory gene expressions extensively depends on changes in external and internal conditions (48). In the natural environment, bacterivorous nematode *C. elegans* encounters different microbes, such as pathogens, predators, competitors, or parasites. Interestingly, with a minimum number of chemosensory neurons, *C. elegans* can navigate and survive in its predominantly microbial natural environment (48). Chemosensory neurons involved in behavioral plasticity of *C. elegans* have been studied (49, 50), but how certain microbes or their secretory product can alter developmental plasticity after being recognized by specific neurons needs more study. Our earlier study demonstrated a negative regulation of *fepB* mutant *Salmonella* infection to develop dauer larvae through activating the TGF-β signaling pathway in the second generation of *C. elegans* population (10). Often, these dauer larvae provide an avoidance strategy and are generationally transmitted to progeny after repeated exposure to bacteria, where escaping is impossible (44). In most cases, the pathogen colonizes the intestine of *C. elegans*, causing a persistent infection that triggers *C. elegans* to enter a dauer state. *P. aeruginosa* PAO1, *S*. Typhimurium MST1, a moderately virulent strain, induced dauer larvae formation, whereas *P. aeruginosa* PA14, a highly virulent strain causing infection to various organisms, led to the killing of *C. elegans* at initial exposure (30, 44); even bacterial small RNA is required for dauer induction, offering mechanistic insight into microbial modulation of nematode development (32). Molecular mechanisms behind dauer larvae development are well defined, but how pathogen infection modulates dormancy in *C. elegans* needs more study (2, 51). Sensory neurons are also involved in dauer decision-making and altering the *C. elegans* lifecycle (4). However, the involvement of nematodes’ chemosensation in altering physiology under the same infection conditions has not been explored extensively. Thereafter, when we exposed olfactory defective mutant *C. elegans* under continuous infection for 8 days (F2 generation) to check the dauer larva formation, we discovered significantly fewer dauer larvae in the nematodes’ total population having defects in AWC neurons than in other strains under the Δ*fepB* strain, but did not alter the growth of the mutant nematodes (Fig. 5A and B). Overall, our study deciphered a significant involvement of AWC chemosensory in recognizing *Salmonella* strains and modulating nematodes’ developmental plasticity. This study will help to illustrate our knowledge in the field of *Salmonella* pathogenesis and host response, where we speculate on the negative regulation of *Salmonella* pathogenesis by the *fepB* gene. Behavioral and metabolic changes in organisms are well-known forms of plasticity that can withstand harsh environmental conditions (2). Pathogens also act as stress boosters for inducing dauer larvae in *C. elegans*. However, some questions still need to be addressed that we were not able to answer in the current study, and further study is required to understand whether the FepB protein (directly or indirectly) or other bacterial secretory molecules are involved in altering the nematodes’ plasticity during the mutant infection, which is kept in check compared to the WT-STM. Besides, detecting the volatile odor secreted by the *fepB* mutant *Salmonella* strain could be an important aspect to see the importance of the molecule to aid AWC-mediated chemosensation in *C. elegans*, and understanding the sensing mechanisms underlying whether the AWC neuron becomes AWCON or AWCOFF to recognize the Δ*fepB* strain secreted odor.

## MATERIALS AND METHODS

### Bacterial strains and growth conditions

*E. coli* strain OP50 (*E. coli* OP50) was a kind gift from Dr. Varsha Singh, Molecular Reproduction, Development and Genetics, IISc. Bangalore, India. *E. coli* OP50 was grown in Luria Bertani (LB) broth and agar and maintained at 37°C. Wild-type *S*. Typhimurium 14028 (WT-STM) was a kind gift from Prof. Dipshikha Chakravortty, Microbiology and Cell Biology Department, IISc. Bangalore, India. Δ*fepB* and Δ*fepB*_c_ were generated in the lab and explained in a previous study and grown in LB broth and agar with specific antibiotics, e.g., Kanamycin (50 µg/mL) and Ampicillin (50 µg/mL; HiMedia laboratory, Mumbai, India) and kept at 37°C.

### *C. elegans* strains and their maintenance

*C. elegans* N2 Bristol Wild Type, CX4 (odr-7[ky4] X), AWC ablated *C. elegans* strains were a kind gift from Dr. Varsha Singh, MRDG, IISc Bangalore; CX2205 (odr-3[n2150] V) mutant was purchased from Caenorhabditis Genetics Center, USA. FK311 (ceh-36[ks86] X), RB2464 (tax-2[ok3403] I), and VC3113 (tax-4[ok3771] III) were a kind gift from Dr. Kavita Babu, Center for Neuroscience, IISc Bangalore, India. Nematodes were maintained at 22°C on NGM seeded with regular laboratory food, i.e., *E. coli* OP50.

### *C. elegans* infection and dauer larvae detection

For the *C. elegans* infection and dauer larvae detection assay, 10 L4 nematodes (P0) were transferred to an NGM plate seeded with *E. coli* OP50, WT-STM, mutant strains, and kept for 8 days at 22°C. On day 8, (F2 generation), each plate was washed three times with M9 buffer, and then after the final wash, the supernatant was discarded, and 1 mL of 1% SDS (dauer larvae were resistant to SDS due to thick cuticle present in their body; HiMedia laboratory, Mumbai, India) was added to 15 mL centrifuge tube and kept under shaking conditions for 30 minutes. Again, nematodes were washed with M9 buffer thrice at 16.5 g for 1 minute and 11.5 g for 3 minutes. During the final wash, sediment larvae were suspended with an equal volume of 60% sucrose. Alive *C. elegans* will come up to the upper layer, and again, they were washed three times with M9 buffer; during the final washing, 90 µL of supernatant was transferred to NGM plates seeded with *E. coli* OP50 and kept for 12–24 hours at 22°C for their recovery.

### Quantification of dauer larva and generation study

The total population of nematodes in percentage was calculated by first collecting the *C. elegans* in 500 µL of M9 buffer. Then, it was diluted in a 1:10 ratio with M9 buffer and counted manually by placing 10 µL of the sample on clean, grease-free slides under a bright-field microscope. Here, the total nematode population in infection plates was normalized with the total population under the *E. coli* OP50 condition. The percentage of dauer larvae was calculated by manually counting the recovered nematodes on each NGM plate seeded with *E. coli* OP50 after 12–24 hours of incubation at 22°C, observed under a microscope with respect to the total population. This experiment represents three independent biological replicates with three technical replicates, and the number of *C. elegans* taken for the experiment was 10 for each condition. The result significance was quantitated using a paired *t-*test and one-way ANOVA by using Bonferroni’s multiple comparison test in GraphPad Prism 5.0 software, as *P*-value < 0.05 ∗, *P*-value < 0.005 ∗∗, and *P*-value < 0.0001 ∗∗∗, with ns-non-significant. Values expressed as mean ± SEM.

### Bacterial food choice assay

Overnight bacterial cultures were resuspended in LB broth at O.D. 0.3. Now, 25 µL of WT-STM/Δ*fepB*/Δ*fepB*_c_ bacterial suspensions were spotted at one side of a 50 mm NGM plate with *E. coli* OP50 on the other side and incubated at 37°C for 12 hours. Fifty to one hundred 1-day adult (N2) *C. elegans* were washed three times with M9 (composition for 1 L: KH_2_PO_4_, 3 g; Na_2_HPO_4_, 6 g; NaCl, 5 g; 1M MgSO_4_, 1 mL; HiMedia laboratory, Mumbai, India) buffer and placed at the center of the assay plates. *C. elegans* were allowed to roam for 1–2 hours. Then, 5 µL of 1 M sodium azide (HiMedia laboratory, Mumbai, India) was added to each bacterial patch to immobilize nematodes and check their food preference using the following choice index equation (5). This experiment represents three independent biological replicates with three technical replicates. The number of *C. elegans* taken for each experiment was 50–100 for each condition. The result significance was quantified using a paired *t*-test in GraphPad Prism 5.0 software, as *P*-value < 0.05 ∗, *P*-value < 0.005 ∗∗, and *P*-value < 0.0001 ∗∗∗, with ns-non-significant. Values expressed as mean ± SEM.


$$Choice\;Index\;(C.I.)=\frac{no.of\;nematodes\;in\;WT-STM\;or\;\Delta \;fepB\;or\;\Delta \;fepBc\;-no.of\;nematodes\;in\;E.coli\;OP50}{Total\;no.of\;nematodes\;on\;both\;bacterial\;patches}.$$

### Aversion response assay

Fifty microliter (O.D. 0.3) of WT-STM/Δ*fepB*/Δ*fepB_c_* bacterial suspensions were spotted in the middle of the 50 mm NGM plate and incubated at 37°C for 12 hours. One hundred one-day adult N2 and olfactory neuron-defective *C. elegans* were washed thrice with M9 buffer and placed at the center of the assay plates. Aversion response was monitored at different time intervals (12, 24, 36, and 48 hours post-infection). Data were plotted based on their position in bacterial lawns and calculated using the following (%) lawn occupancy equation. This experiment represents three independent biological replicates with three technical replicates, and the number of *C. elegans* taken for each experiment was 100 for each condition. The result significance was quantified using one-way ANOVA using Bonferroni’s multiple comparison test in GraphPad Prism 5.0 software, as *P*-value < 0.05 ∗, *P*-value < 0.005 ∗∗, and *P*-value < 0.0001 ∗∗∗, with ns-non-significant. Values expressed as mean ± SEM.


$$\%\;Lawn\;occupancy\;=\;\;\frac{no.\;of\;nematodes\;inside\;the\;lawn}{Total\;no.\;of\;nematodes}*100.$$

### Olfactory aversive learning assay

Around 200 age-synchronized L4 stage N2 and olfactory neuron defective *C. elegans* were transferred to an NGM plate seeded with *E. coli* OP50 (untrained plate), and 100 *C*. *elegans* were transferred to four different NGM plates seeded at one corner with 50 µL of *E. coli* OP50 and 200 µL of WT-STM/Δ*fepB*/Δ*fepB_c_* strains to another corner (trained plate). After 48 hours of incubation at 22°C, 30 *C*. *elegans* from trained and untrained plates are transferred to each 50 mm assay plate containing 50 µL of *E. coli* OP50 and WT-STM/Δ*fepB*/Δ*fepB_c_* strains and observed after 2 hours of incubation. Then, 5 µL of 1 M sodium azide (HiMedia laboratory, Mumbai, India) was added to each bacterial patch to immobilize nematodes and check their food preference and aversive learning behavior by using the following choice index and learning index equations (52). This experiment represents three independent biological replicates with three technical replicates, and the number of *C. elegans* taken for each experiment was 30 for each condition. The result significance was quantified using one-way ANOVA with Bonferroni’s multiple comparison test in GraphPad Prism 5.0 software, as *P*-value < 0.05 ∗, *P*-value < 0.005 ∗∗, and *P*-value < 0.0001 ∗∗∗, ns-non-significant. Values expressed as mean ± SEM.


$$Choice\;Index\;\left(C.I.\right)\;=\;\;\frac{no.\;of\;nematodes\;in\;WT-STM\;or\;\Delta fepB-no.\;of\;nematodes\;in\;E.\;coli\;OP50}{Total\;no.\;of\;nematodes\;on\;both\;bacterial\;patches\;}$$

Learning Index (L.I.) = nematodes in naïve plate – nematodes in trained plate

### Quantitative real-time PCR

RNA from 12- to 48-hour-infected *C. elegans* was extracted using TRIzol reagent (Invitrogen, California, USA), per the manufacturer’s instructions. After the quality check, DNase (Promega, Madison, USA) treatment, and control PCR, 2 µg of RNA was used for cDNA synthesis by Go Script TM Reverse Transcription System (Promega, Madison, USA). Then, quantitative real-time PCR was performed using Maxima SYBR Green/ROX qPCR master mix (Thermo Scientific, Waltham, USA) with gene-specific primers (Sigma, Bangalore, India; Tables 1 and 2) in Realplex4 Eppendorf system. Relative expression of the gene was calculated using the 2^−ΔΔCT^ method and plotted as fold change (53). This experiment represents three independent biological replicates with three technical replicates, and 1,500–2,000 *C. elegans* were taken per condition for RNA isolation. Significance was quantified using one-way ANOVA by using Bonferroni’s multiple comparison test in GraphPad Prism 5.0 software, *P*-value < 0.05 ∗, *P*-value < 0.005 ∗∗, and *P*-value < 0.0001 ∗∗∗, with ns-non-significant. Values expressed as mean ± SEM.

**TABLE 1:** List of primers used in the study

Primer	Forward primer (5′−3′)	Reverse primer (5′−3′)
*ama-1*	CCCGGAGGAGATTAAACG	CATGTCATGCATCTTCCAC
*odr-10*	CAAACGCCAATTTCATTGTG	CATCACGTCGGAACTTGAGA
*odr-3*	TTCTCGAGTGGCAACAACTG	ACTCGATCATTCGGTTCGTC
*ceh-36*	ACGGCACCCAGTCTTACATC	GTGCACATCCGGATACTGTG
*tph-1*	CGCCGGATACTTATCAGCTC	CAGCGAATAGAGCCATGTGA
*tax-2*	ATCCGTGCAAAGGGTTACTG	TGCTTGCGCCTTTATCTTTT
*tax-4*	CATACGACTACGGCTCAGCA	CGTATCCGAACCGCATAACT
*ocr-2*	CGACTCCAAAAAGGGACAGA	TCAGTGAGCAGCACCATTTC
*osm-9*	AGCTTCGATGGGTGGTTATG	CGATAACCGTCGTGTACGTG

**TABLE 2:** List of olfactory chemosensory neuron-specific genes with function used in the study

Neurons	Functions	Major G protein receptor/transcription factor	Signal transduction
AWA	Volatile chemotaxis	*odr-10* and *odr-3*	*osm-9* and *ocr-2*
AWB	Volatile avoidance	*odr-3*	*tax-4*, *tax-2*, and *daf-11*
AWC	Volatile chemotaxis	*ceh-36*	*tax-4*, *tax-2*, and *daf-11*

### Statistical analysis

The results obtained are represented statistically as mean ± SEM. For multiple samples, data were analyzed using a paired *t*-test and one-way ANOVA, followed by the Bonferroni multiple comparisons test. To analyze the statistically significant results, we considered a *P*-value less than 0.05 statistically significant.

## SUPPLEMENTAL MATERIAL

Figures S1 and S2 spectrum.00420-25-s0001.docx Fig. S1: ΔfepB *Salmonella* strain altered olfactory chemosensory response in *C. elegans*. Fig. S2: ΔfepB *Salmonella* strain alters olfactory chemosensory response in *C. elegans*.

ASM does not own the copyrights to Supplemental Material that may be linked to, or accessed through, an article. The authors have granted ASM a non-exclusive, world-wide license to publish the Supplemental Material files. Please contact the corresponding author directly for reuse.
