# Supplementary material for: Caenorhabditis elegans AWC neuron-mediated chemosensation negatively modulates dormancy during Salmonella fepB mutant infection
Source: Microbiol Spectr. 2025 Oct 8;13(11):e00420-25. doi: 10.1128/spectrum.00420-25 (PMC12584732; doi:10.1128/spectrum.00420-25)
Supplement: Figures S1 and S2 — Fig. S1: ΔfepB Salmonella strain altered olfactory chemosensory response in C. elegans. Fig. S2: ΔfepB Salmonella strain alters olfactory chemosensory response in C. elegans. [file spectrum.00420-25-s0001.docx]

**Supplemental Figures and legends:**


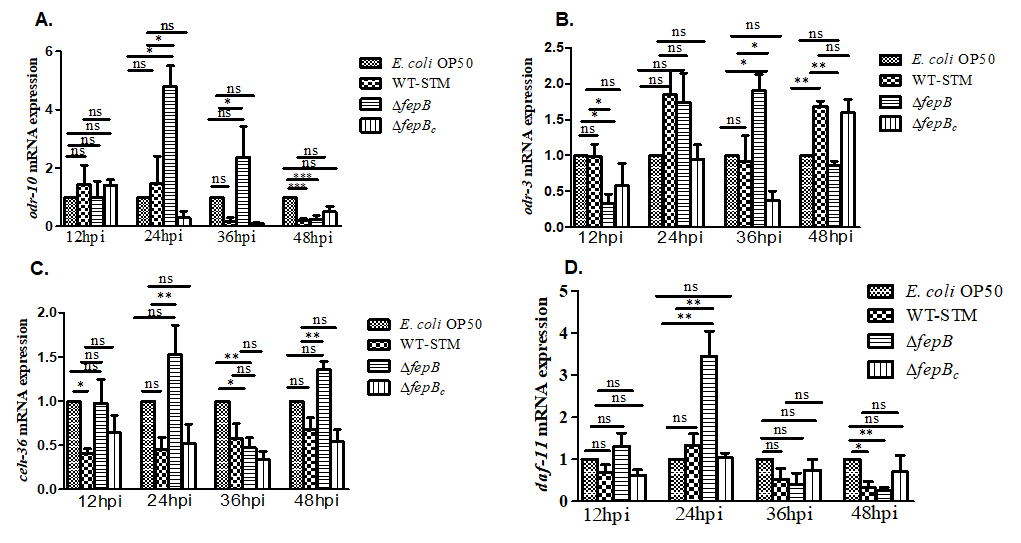


**Fig. S1.** **Δ*fepB Salmonella* strain altered olfactory chemosensory response in *C. elegans*.** A-D. Age-synchronized L4 *C. elegans* larvae were kept in *E. coli* OP50, plate WT-STM/ Δ*fepB/* Δ*fepB_c_* for 12, 24, 36, and 48 hours. *C. elegans* were taken for RNA isolation, and after quality and quantity checking, qRT-PCR was performed for olfactory neuron-specific genes, i.e., *odr-10, odr-3, ceh36, daf-11*; *ama-1* was used as the internal control, and relative fold changes were calculated using the comparative 2^ΔΔCT^ method. Data represented three biological replicates and three technical replicates; result significance was quantified as *p*-value < 0.05 ∗, *p*-value < 0.005, ∗∗and *p*-value < 0.0 0 01 ∗∗∗, ns-non-significant. Values expressed as Mean ±SEM.


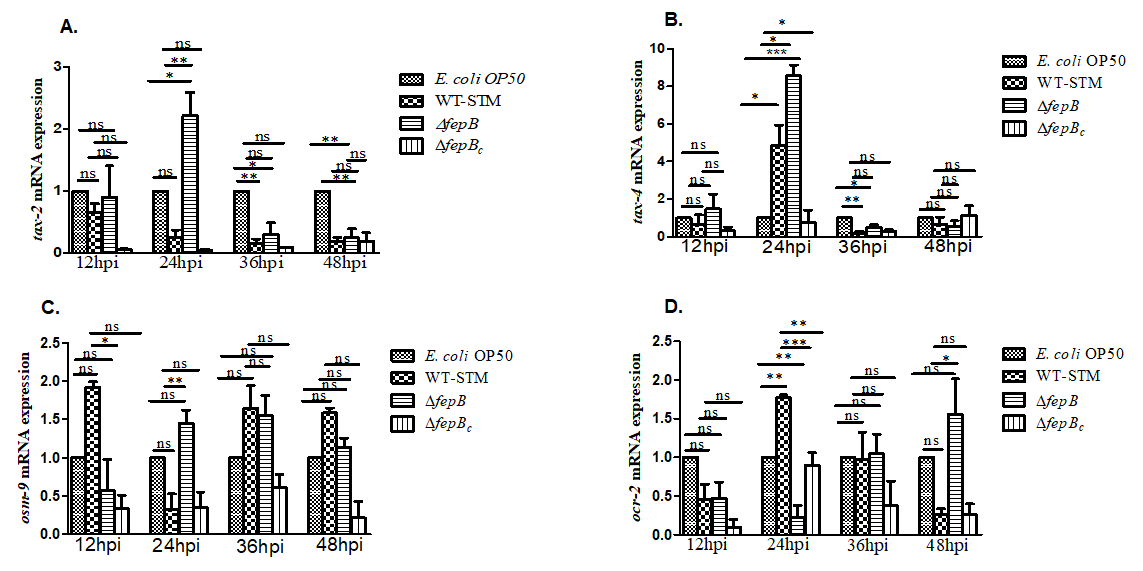


**Fig. S2.** **Δ*fepB Salmonella* strain alters olfactory chemosensory response in *C. elegans****.* A-D. Age-synchronized L4 *C. elegans* larvae were kept in *E. coli* OP50, plate WT-STM/ Δ*fepB/* Δ*fepB_c_* for 12, 24, 36, and 48 hours. *C. elegans* were taken for RNA isolation, and after quality and quantity checking, qRT-PCR was performed for olfactory neuron-specific genes, i.e., *tax-2, tax-4, osm-9, ocr-2*; *ama-1* was used as the endogenous control, and relative fold changes were calculated using the comparative 2^ΔΔCT^ method. Data represented three biological replicates and three technical replicates; result significance was quantified as *p*-value < 0.05 ∗, *p*-value < 0.005, ∗∗and p-value < 0.0 0 01 ∗∗∗, ns-non-significant. Values expressed as Mean ±SEM.
